# [Retracted] Anti‑carcinogenic activity of anandamide on human glioma *in vitro* and *in vivo*

**DOI:** 10.3892/mmr.2023.12969

**Published:** 2023-02-28

**Authors:** Chao Ma, Ting-Ting Wu, Pu-Cha Jiang, Zhi-Qiang Li, Xin-Jun Chen, Kai Fu, Wei Wang, Rui Gong

Mol Med Rep 13: 1558–1662, 2016; DOI: 10.3892/mmr.2015.4721

Following the publication of this paper, it was drawn to the Editors’ attention by a concerned reader that certain of the scratch-wound data shown in Fig. 3A were strikingly similar to data appearing in different form in another article by different authors. Owing to the fact that the contentious data in the above article had already been published elsewhere prior to its submission to *Molecular Medicine Reports*, the Editor has decided that this paper should be retracted from the Journal. The authors were asked for an explanation to account for these concerns, but the Editorial Office did not receive a reply. The Editor apologizes to the readership for any inconvenience caused.

